# Supplementary material for: Multiparametric senescent cell phenotyping reveals targets of senolytic therapy in the aged murine skeleton
Source: Nat Commun. 2023 Jul 31;14:4587. doi: 10.1038/s41467-023-40393-9 (PMC10390564; doi:10.1038/s41467-023-40393-9)
Supplement: Supplementary file 1 — Supplementary Information [file 41467_2023_40393_MOESM1_ESM.pdf]

Supplementary Information

Multiparametric senescent cell phenotyping reveals targets of senolytic therapy in the aged murine skeleton

Madison L. Doolittle[1,2], Dominik Saul[1,2,3], Japneet Kaur[1,2], Jennifer L. Rowsey[1,2], Stephanie J. Vos[1,2], Kevin D. Pavelko[4], Joshua N. Farr[1,2], David G. Monroe[1,2], and Sundeep Khosla, M.D*.[1,2]

[1]Division of Endocrinology, Diabetes and Metabolism, Mayo Clinic, Rochester, MN, 55905, USA.

[2]Robert and Arlene Kogod Center on Aging, Mayo Clinic, Rochester, MN 55905, USA.

[3]Department for Trauma and Reconstructive Surgery, BG Clinic, University of Tübingen, Germany

[4]Department of Immunology, Mayo Clinic, Rochester, MN, 55905, USA.

***Correspondence:** Sundeep Khosla, M.D., Guggenheim 7-11, Mayo Clinic College of Medicine, 200 First Street SW, Rochester, MN 55905; Tel: +1-507-255-6663; Email: khosla.sundeep@mayo.edu

This PDF file includes:

Supplementary Figures 1-9

Supplementary Tables 1-3

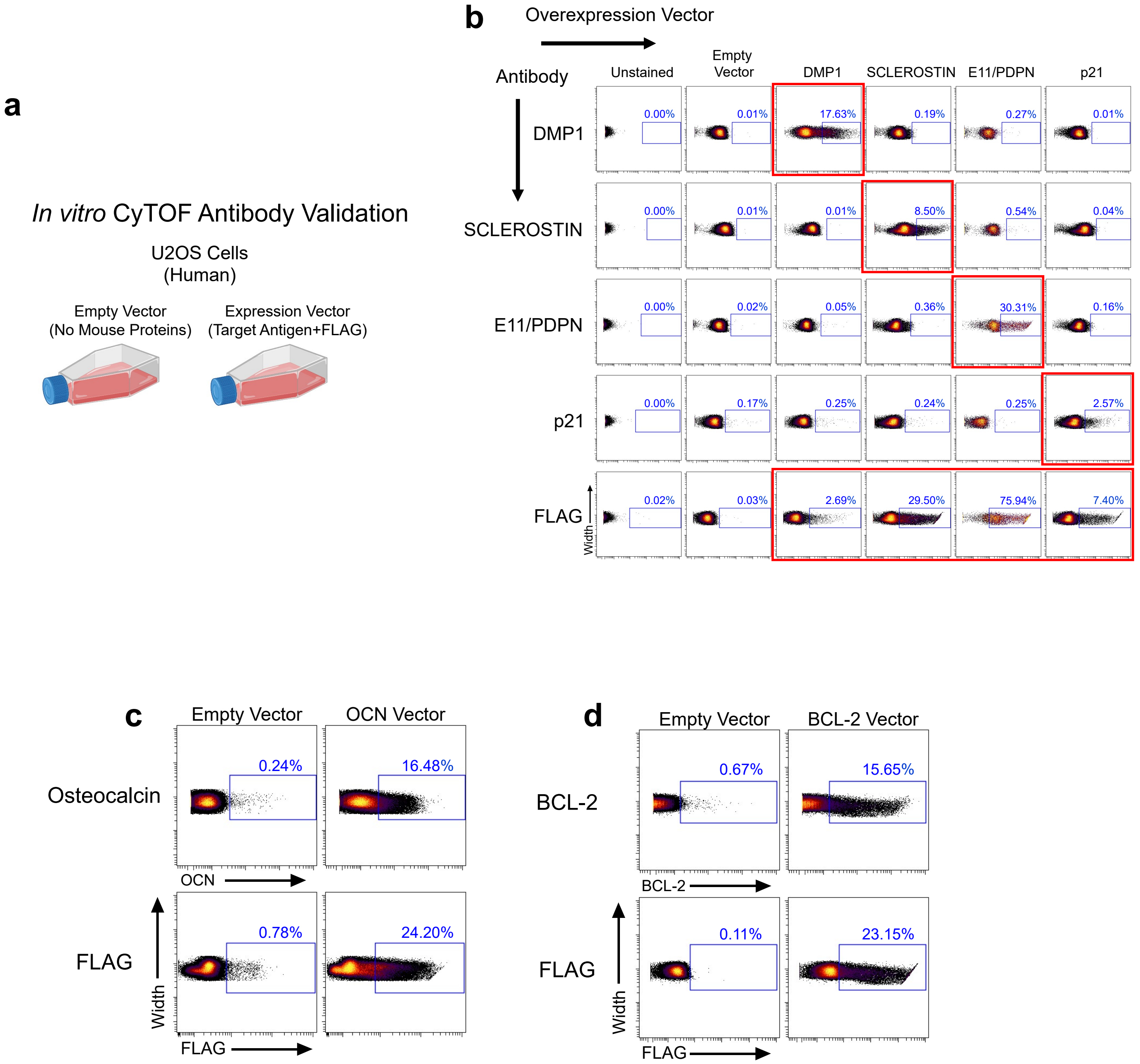

Supplementary Figure 1. Validation of antibodies in CyTOF panel. (a) Experimental workflow of single mouse protein expression in U2OS cells for the testing of CyTOF antibodies; (b) CyTOF plots of several expression tests run simultaneously. Each column is an individual sample, and each row is the antibody being tested. Red boxes demonstrate positive results, where signal is observed in the positive gate (blue box) and not observed in any other channel or sample. FLAG demonstrates successful expression of the DNA plasmid; (c,d) Additional validations of osteocalcin and BCL-2 antibodies by FLAG-tagged expression vectors. Schematic in (a) was generated using BioRender. Source data are provided as a Source Data file.

a

Sample	Live Cells	Viability	Singlets	CD45-
Young Non-Digested	208,780	95.71%	107,001	3,537
Young Digested	292,649	95.94%	170,988	15,817
Old Non-Digested	196,045	94.49%	95,721	5,004
Old Digested	358,150	95.82%	191,297	7,099

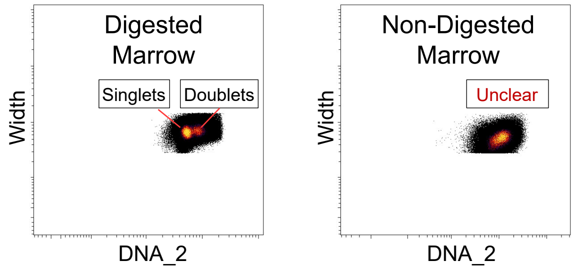

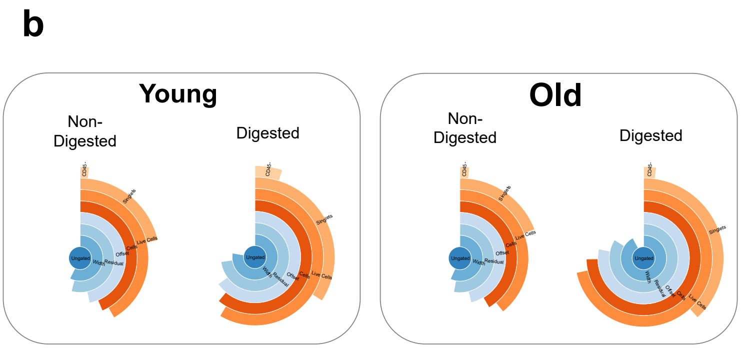

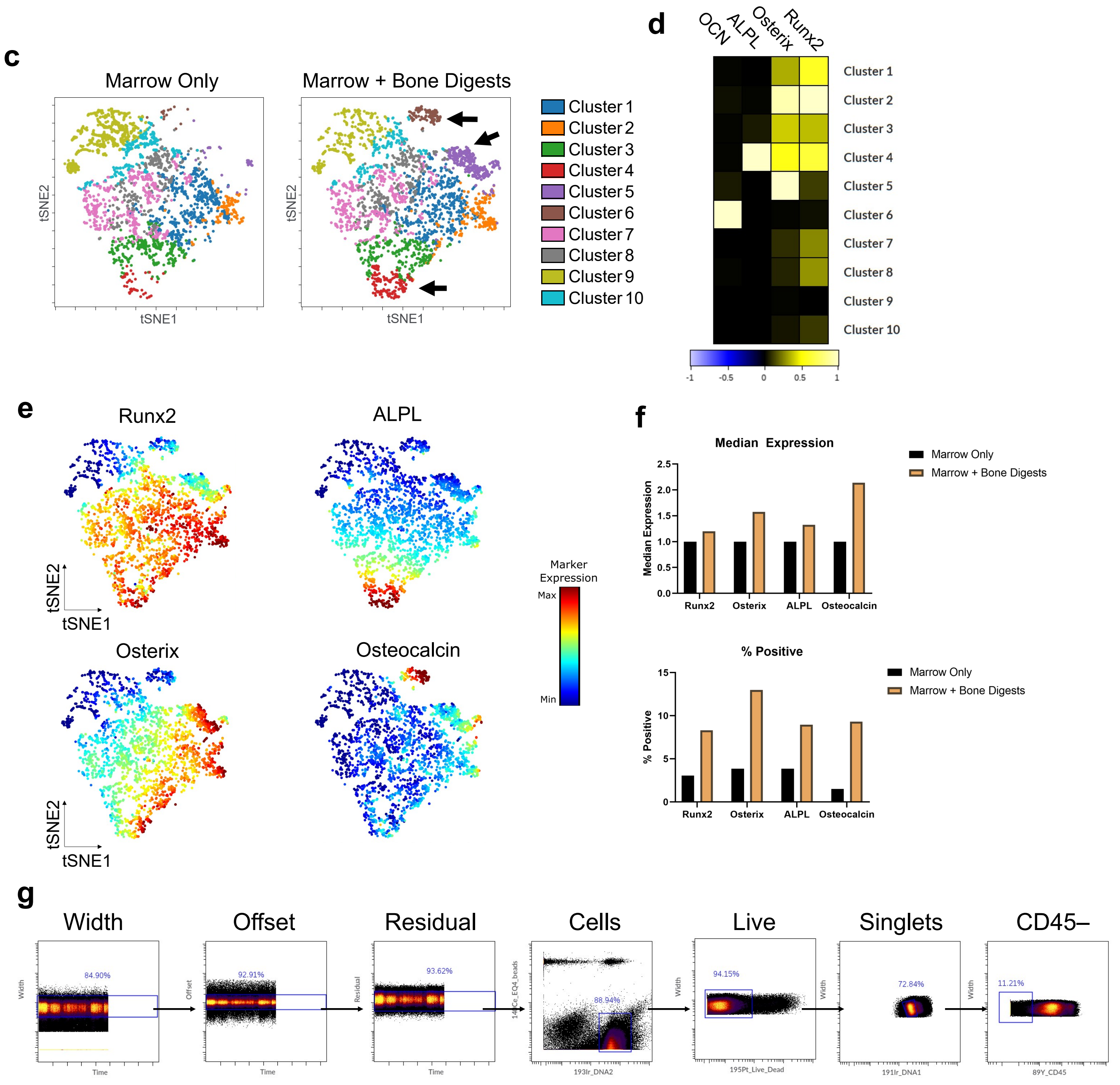

Supplementary Figure 2. Optimization of bone and marrow digestion protocols. (a) Quantification of results and CyTOF plots demonstrating better resolution of singlets and doublets with Liberase digestion (X-axis is DNA content, while Y-axis is width). (b) Sunburst plots from experiments trialing the Liberase digestion of bone marrow samples, demonstrating an increase in yield of total and CD45- cells, with a preservation in cell viability; (c) t-SNE plots of samples containing cells from either marrow-only or marrow combined with 3 bone digestions. FlowSOM-clustered cell populations demonstrate an emergence of several clusters, marked by arrows, when adding in cells isolated from digested bone; (d) Heatmap of median expression and (e) feature plots of osteolineage markers across clusters, demonstrating expression of ALPL, Osterix, and Osteocalcin (OCN) in emerging clusters 4, 5, and 6, respectively; (f) Overall median expression and percent-positive values of osteolineage markers, demonstrating an enrichment with cells obtained from digested bone. n=1 mouse per condition. (g) Gating strategy for cleanup and purification of mesenchymal cells from digested bone/bone marrow cell suspensions. Source data are provided as a Source Data file.

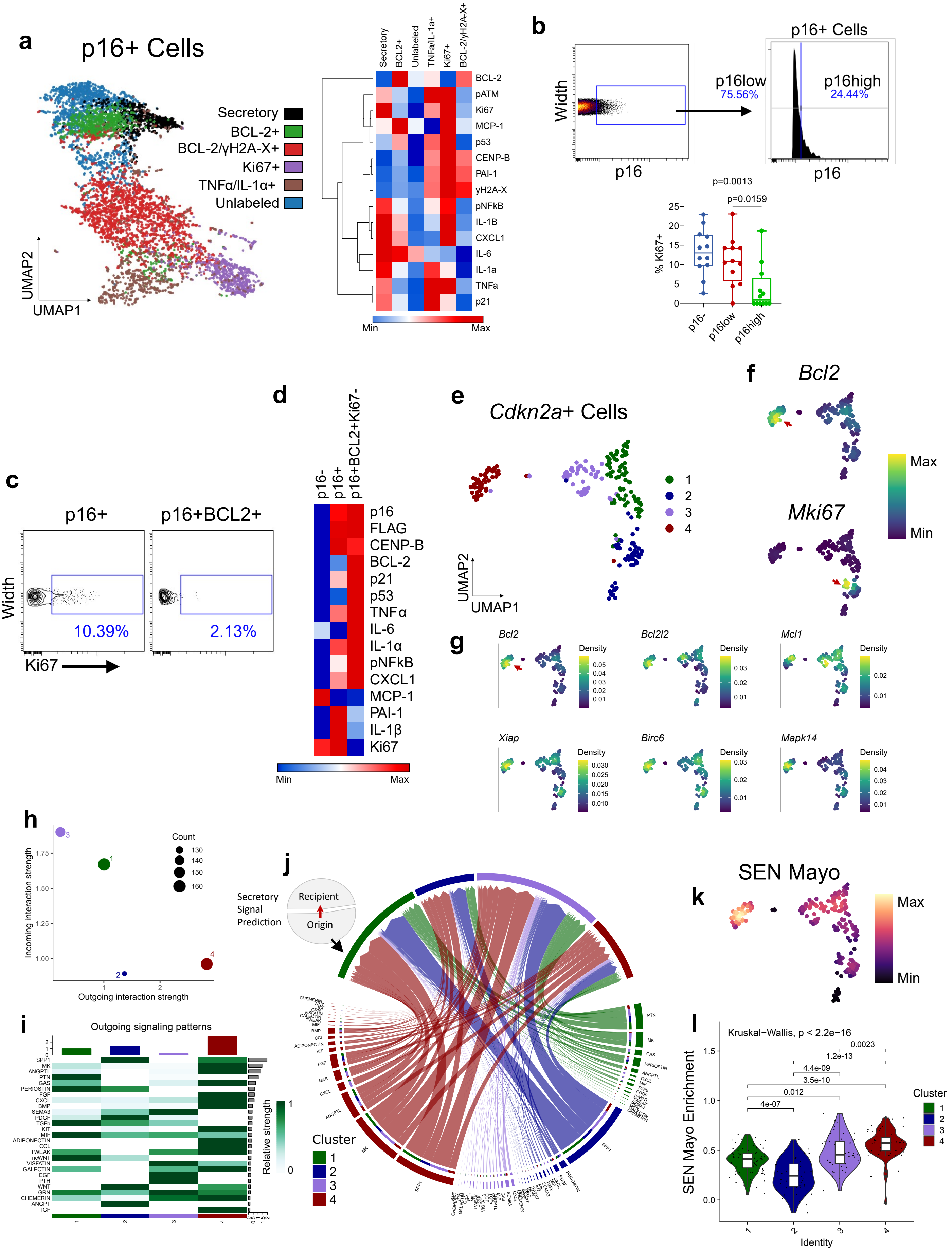
a
p16+ Cells
Secretory
BCL-2+
BCL-2/γH2A-X+
Ki67+
TNFα/IL-1α+
Unlabeled
UMAP2
UMAP1
Secretory
BCL2+
Unlabeled
TNFa/IL-1a+
Ki67+
BCL-2/γH2A-X+
BCL-2
pATM
Ki67
MCP-1
p53
CENP-B
PAI-1
γH2A-X
pNFkB
IL-1B
CXCL1
IL-6
IL-1a
TNFa
p21
Min
Max
b
Width
p16
p16low
75.56%
p16+ Cells
p16high
24.44%
p16
p=0.0013
p=0.0159
% Ki67+
p16-
p16low
p16high
c
p16+
p16+BCL2+
Width
10.39%
2.13%
Ki67
d
p16-
p16+
p16+BCL2+Ki67-
p16
FLAG
CENP-B
BCL-2
p21
p53
TNFα
IL-6
IL-1α
pNFkB
CXCL1
MCP-1
PAI-1
IL-1β
Ki67
Min
Max
e
Cdkn2a+ Cells
UMAP2
UMAP1
f
Bcl2
Mki67
Max
Min
g
Bcl2
Bcl2l2
Mcl1
Xiap
Birc6
Mapk14
Density
h
Count
Incoming interaction strength
Outgoing interaction strength
i
Outgoing signaling patterns
Relative strength
j
Secretory Signal Prediction
Recipient
Origin
Cluster
k
SEN Mayo
Max
Min
l
Kruskal–Wallis, p < 2.2e−16
SEN Mayo Enrichment
Identity
Cluster

Supplementary Figure 3: (a) UMAP visualization and FlowSOM clustering of p16+ cells from young and old mice (merged) with corresponding heatmap demonstrating senescence panel marker expression in the 6 identified clusters; (b) Gating strategy for p16low and p16high cells, with %Ki67+ for each population below alongside p16- cells. (c) Dot plots of BCL2+ subsetting of p16+ cells demonstrating %Ki67+ cells in each population (blue gate; n=12 old biologically independent animals); (d) Heatmap demonstrating mean expression of SASP markers between p16-, p16+, and p16KB cells; (e) scRNA-seq UMAP visualization of Cdkn2a+ (p16-encoding gene) cells from digested bone and marrow mesenchymal cells; (f) Density expression plots of Bcl2 and Mki67 gene expression within Cdkn2a+ cells; (g) Expression plots of apoptosis resistance genes; (h) Incoming and outgoing secretory interaction strength predictions of clusters within Cdkn2a+ cells; (i) Network centrality scores of outgoing signaling patterns in Cdkn2a+ cells; (j) Chord diagram demonstrating all outgoing and incoming signaling patterns within Cdkn2a+ cells; (k) Enrichment score for SenMayo geneset demonstrated over UMAP visualization and (l) corresponding quantification within Cdkn2a+ cell clusters (each dot represents one cell; n=2 biologically independent animals). Box plots show median and interquartile range with error bars representing minimum and maximum values. (b) One-way ANOVA with Tukey’s Multiple Comparisons test. (l) Kruskal-Wallis test with Dunn’s test for pairwise comparisons. Source data are provided as a Source Data file.

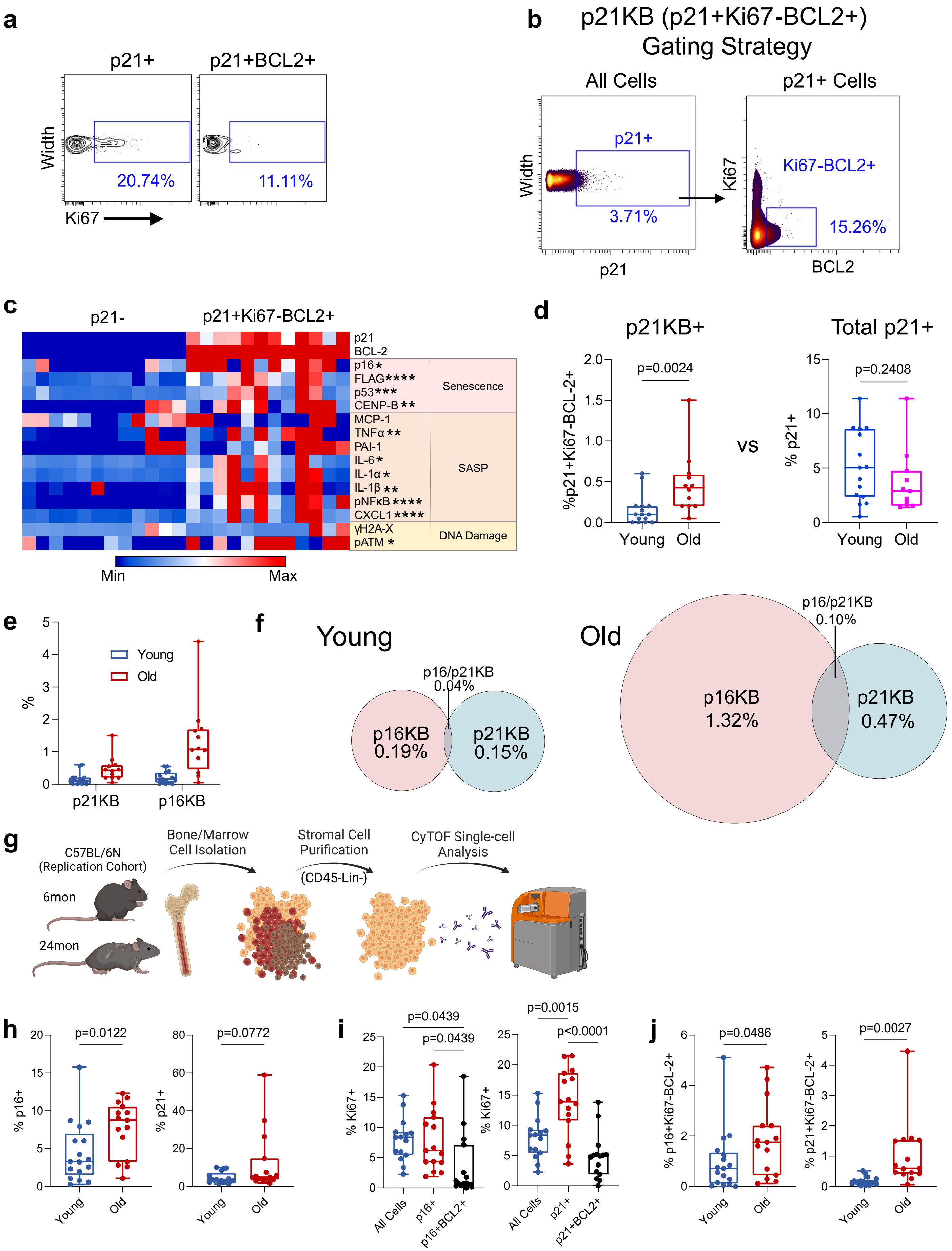

a
p21+
p21+BCL2+
Width
Ki67
20.74%
11.11%
b
p21KB (p21+Ki67-BCL2+)
Gating Strategy
All Cells
p21+ Cells
p21+
3.71%
Width
p21
Ki67
Ki67-BCL2+
15.26%
BCL2
c
p21-
p21+Ki67-BCL2+
p21
BCL-2
p16*
FLAG****
p53***
CENP-B**
MCP-1
TNFα**
PAI-1
IL-6*
IL-1α*
IL-1β**
pNFκB****
CXCL1****
γH2A-X
pATM *
Senescence
SASP
DNA Damage
Min
Max
d
p21KB+
Total p21+
p=0.0024
p=0.2408
%p21+Ki67-BCL-2+
% p21+
vs
Young
Old
e
Young
Old
%
p21KB
p16KB
f
Young
Old
p16/p21KB
0.04%
p16KB
0.19%
p21KB
0.15%
p16/p21KB
0.10%
p16KB
1.32%
p21KB
0.47%
g
C57BL/6N
(Replication Cohort)
6mon
24mon
Bone/Marrow
Cell Isolation
Stromal Cell
Purification
(CD45-Lin-)
CyTOF Single-cell
Analysis
h
p=0.0122
p=0.0772
% p16+
% p21+
Young
Old
i
p=0.0439
p=0.0439
p=0.0015
p<0.0001
% Ki67+
All Cells
p16+
p16+BCL2+
p21+
p21+BCL2+
j
p=0.0486
p=0.0027
% p16+Ki67-BCL-2+
% p21+Ki67-BCL-2+

Supplementary Figure 4. Defining growth-arrested subset of p21+ cells associated with age and replication of BCL-2 results. (a) Dot plots of BCL2+ subsetting of p21+ cells demonstrating %Ki67+ cells in each population (blue gate); (b) Gating strategy for p21KB cells; (c) Heatmap representation of protein expression between p21KB cells and p21- cells, with asterisks indicating significance (*$p<0.05$, **$p<0.01$, ****$p<0.0001$). (d) Quantification of p21KB cells with age and comparison to total p21+ cells (n=15 young and n=12 old biologically independent animals). (e) Comparison of p21KB vs p16KB cell proportions (n=15 young and n=12 old). (f) Venn diagrams of p16KB versus p21KB cells demonstrating very few shared cells between populations. (g) Schematic of replication cohort workflow, containing n=17 young and n=15 old C57BL/6N mice (Old + Vehicle mice from D+Q experiment); (h) Percent-positive plots of p16+ or p21+ cells between young and old mice. (i) Percent Ki67+ in all cells, p16+/p21+ cells, and BCL-2+ subsets of each. (j) Percent p16KB or p21KB cells with age. Schematic in (g) was generated using BioRender. Box plots show median and interquartile range with error bars representing minimum and maximum values. (c, d, h, j) Two-sided unpaired t test or Mann-Whitney test as appropriate. (i) Multiple two-sided t tests with Holm-Sidak Correction. Source data are provided as a Source Data file.

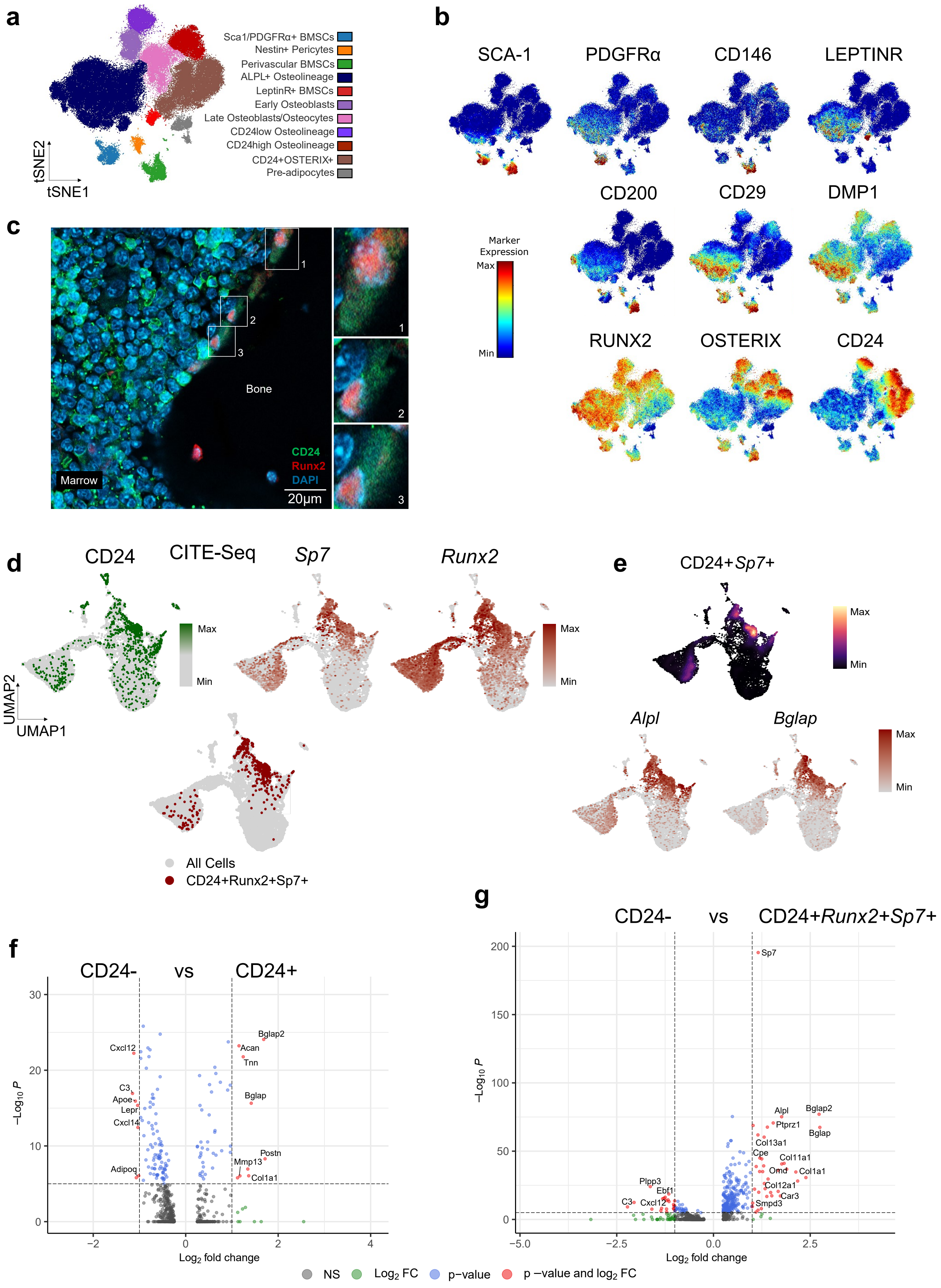
a
Sca1/PDGFRα+ BMSCs
Nestin+ Pericytes
Perivascular BMSCs
ALPL+ Osteolineage
LeptinR+ BMSCs
Early Osteoblasts
Late Osteoblasts/Osteocytes
CD24low Osteolineage
CD24high Osteolineage
CD24+OSTERIX+
Pre-adipocytes
tSNE2
tSNE1
b
SCA-1
PDGFRα
CD146
LEPTINR
CD200
CD29
DMP1
RUNX2
OSTERIX
CD24
Marker Expression
Max
Min
c
Bone
Marrow
CD24
Runx2
DAPI
20μm
1
2
3
d
CD24
CITE-Seq
Sp7
Runx2
Max
Min
UMAP2
UMAP1
All Cells
CD24+Runx2+Sp7+
e
CD24+Sp7+
Alpl
Bglap
f
CD24- vs CD24+
Bglap2
Cxcl12
Acan
Tnn
C3
Apoe
Lepr
Cxcl14
Bglap
Postn
Mmp13
Adipoq
Col1a1
Log2 fold change
−Log10 P
g
CD24- vs CD24+Runx2+Sp7+
Sp7
Alpl
Bglap2
Ptprz1
Bglap
Col13a1
Cpe
Col11a1
Omd
Col1a1
Plpp3
Ebf1
Col12a1
Car3
C3
Cxcl12
Smpd3
NS
Log2 FC
p−value
p −value and log2 FC

Supplementary Figure 5. Characterization of CD24 Osteolineage cells. (a) t-SNE visualization and FlowSOM clustering of CD45-Lin- mouse bone and marrow cells analyzed by CyTOF. (b) t-SNE feature plots depicting protein expression of cluster-defining phenotypic markers; (c) Immunofluorescent staining of CD24 on femur section from Runx2-Cre x TdTomato mouse line demonstrating co-expression of CD24 and Runx2 on bone-lining cells, magnified (results from n=1 experiment); (d) UMAP feature plots of CD24 protein alongside Runx2 and Sp7 mRNA in bone and marrow cells analyzed by CITE-Seq, with CD24 osteolineage (CD24+Runx2+Sp7+) cells plotted below; (e) Feature plots of mature (Alpl, Bglap) gene expression alongside CD24+Sp7+ subset. (f) Differential expression of CD24+ or (g) CD24+Runx2+Sp7+ versus CD24- cells (LogFC > 1.0 and adjusted p-value > Log10(10e-6); Wilcoxon Rank Sum test with Bonferonni correction).

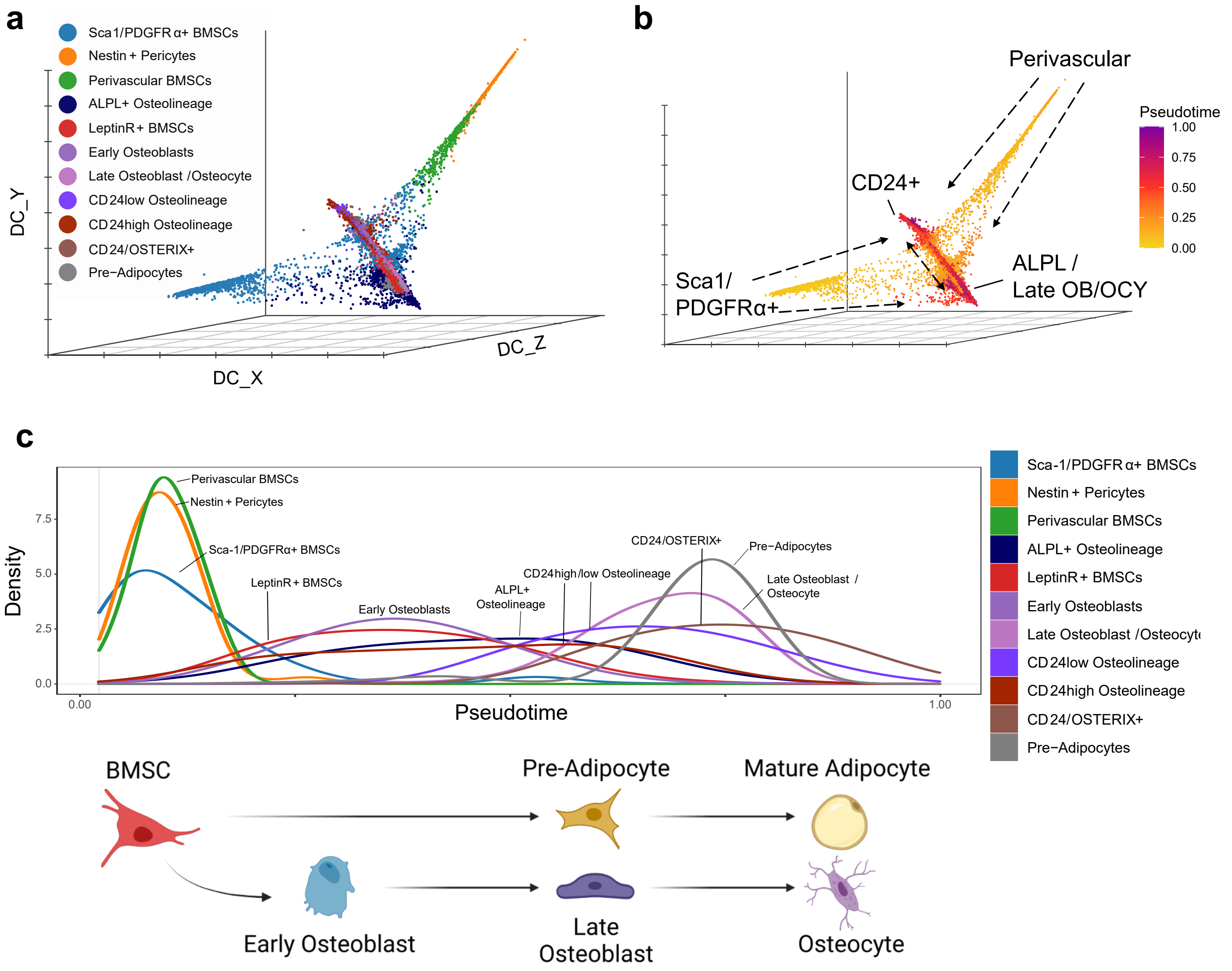

Supplementary Figure 6. Single-cell CyTOF reconstruction of the mesenchymal bone/bone marrow microenvironment. (a) Diffusion map visualization of clustered populations; (b) Pseudotime trajectory displayed on diffusion map displaying CD24 osteolineages (CD24+), ALPL+ osteolineage cells (ALPL), and late osteoblast/osteocyte (Late OB/OCY) clusters as late in differentiation downstream of the Perivascular and Sca1/PDGFRα+ BMSC clusters. Note the divergence of the CD24+ versus the ALPL+ osteolineage and late osteoblast/osteocyte clusters. Dotted arrows depict pseudotime trajectory; (c) Density of clusters along pseudotime consistent with established cell differentiation patterns, shown by the diagram below (generated using BioRender).

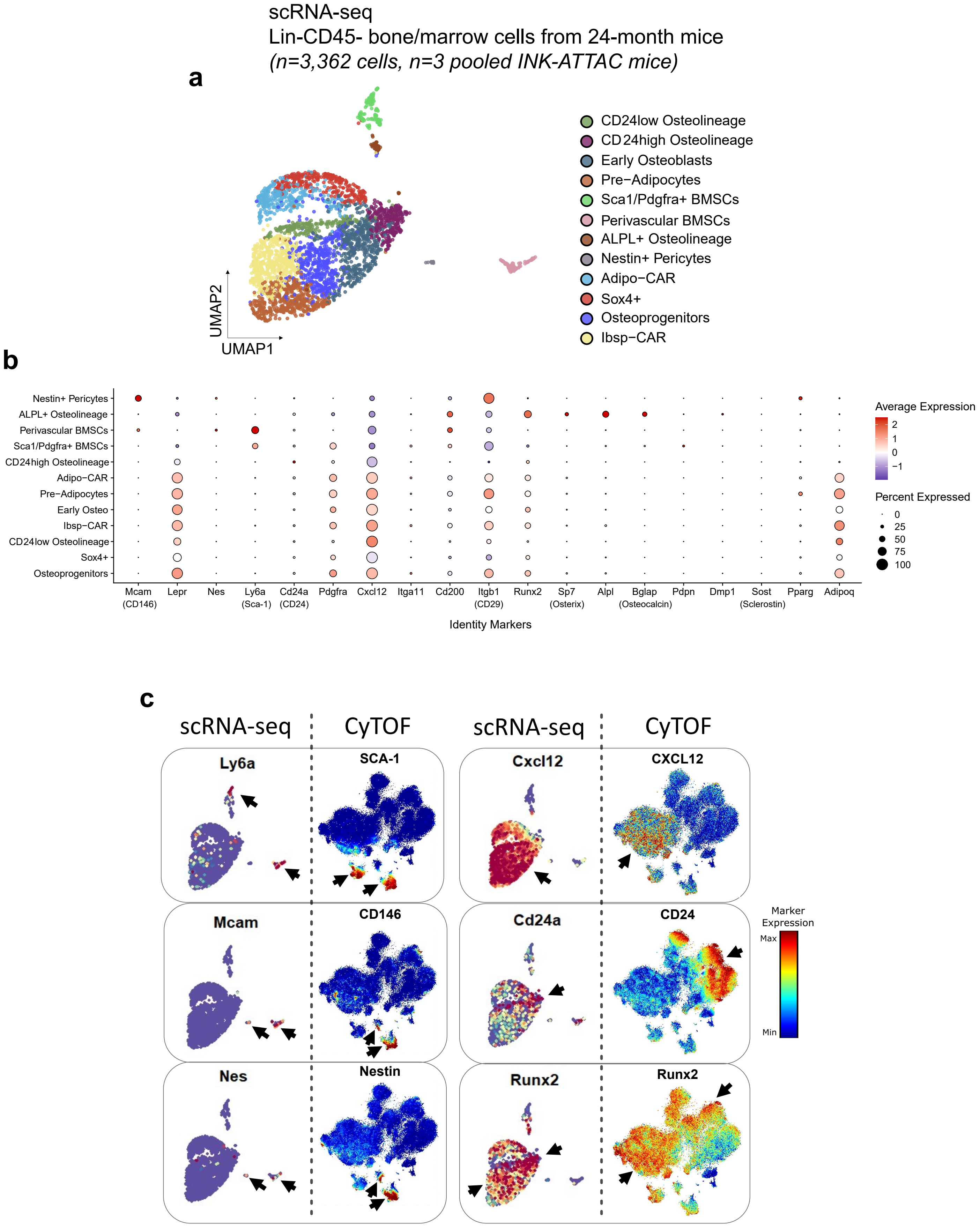

Supplementary Figure 7. Validation of CyTOF populations by scRNA-seq. (a) UMAP visualization of n=3,362 clustered Lin-CD45- cells from the digested bone and marrow of n=3 24-month untreated INK-ATTAC mice; (b) Dot plot expression of markers defining each scRNA-seq cluster; (c) Visual comparison of multidimensional scRNA-seq and CyTOF single-cell data, demonstrating similar expression patterns of common mesenchymal markers using each tool, marked by black arrows.

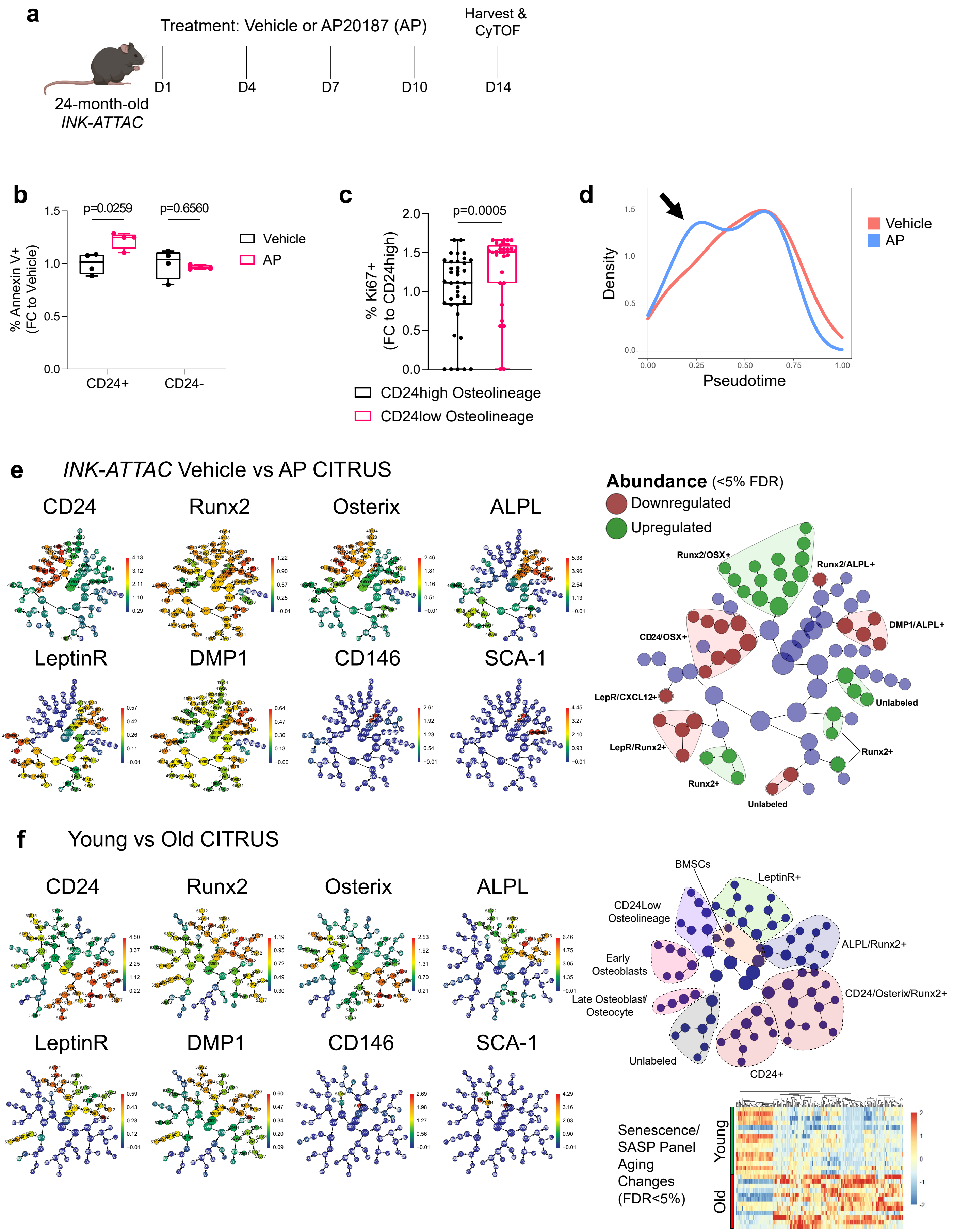
a
Treatment: Vehicle or AP20187 (AP)
Harvest & CyTOF
D1
D4
D7
D10
D14
24-month-old
INK-ATTAC
b
% Annexin V+ (FC to Vehicle)
p=0.0259
p=0.6560
1.5
1.0
0.5
0.0
CD24+
CD24-
Vehicle
AP
c
% Ki67+ (FC to CD24high)
p=0.0005
2.0
1.5
1.0
0.5
0.0
CD24high Osteolineage
CD24low Osteolineage
d
Density
Pseudotime
0.00
0.25
0.50
0.75
1.00
Vehicle
AP
e
INK-ATTAC Vehicle vs AP CITRUS
CD24
Runx2
Osterix
ALPL
LeptinR
DMP1
CD146
SCA-1
Abundance (<5% FDR)
Downregulated
Upregulated
Runx2/OSX+
Runx2/ALPL+
CD24/OSX+
DMP1/ALPL+
LepR/CXCL12+
Unlabeled
LepR/Runx2+
Runx2+
Runx2+
Unlabeled
f
Young vs Old CITRUS
CD24
Runx2
Osterix
ALPL
LeptinR
DMP1
CD146
SCA-1
BMSCs
LeptinR+
CD24Low Osteolineage
ALPL/Runx2+
Early Osteoblasts
CD24/Osterix/Runx2+
Late Osteoblast/ Osteocyte
Unlabeled
CD24+
Senescence/ SASP Panel Aging Changes (FDR<5%)
Young
Old

Supplementary Figure 8. INK-ATTAC Analyses and CITRUS Defining markers. (a) Schematic of p16+ senescent cell clearance in INK-ATTAC mice analyzed by CyTOF. Mice were treated with vehicle or AP every three days for 14 days (indicated by dashes); (b) Percent Annexin V+ cells in freshly isolated INK-ATTAC (n=4) bone and marrow cells treated with AP20187 for 24hrs; (c) Percent Ki67+ in CD24low and CD24high Osteolineage FlowSOM populations from all subgroups; (d) Pseudotime density plot of vehicle- and AP-treated samples, demonstrating a surge in early-pseudotime cell types, marked by black arrow. (e) Expression plots for defining markers of CITRUS cluster families for vehicle- versus AP-treated INK-ATTAC mice. Plots demonstrating cell abundance changes within clusters after AP treatment ($q<0.05$), with red marking cleared clusters and green marking upregulated clusters; (f) Expression plots for defining markers of CITRUS cluster families for INK-ATTAC young (n=15) vs old (n=12) analyses corresponding to Figure 4c. Cluster families are colored and indicated by dotted lines. Heatmap demonstrates all significant (FDR<5%) CITRUS results of senescence panel changes with age: Rows are mice and each column is a cluster-marker combination that reached significance. Schematic in (a) was generated using BioRender. Box plots show median and interquartile range with error bars representing minimum and maximum values. (b) Multiple two-sided t-tests with Holm-Sidak correction, (c) two-sided Mann-Whitney test.

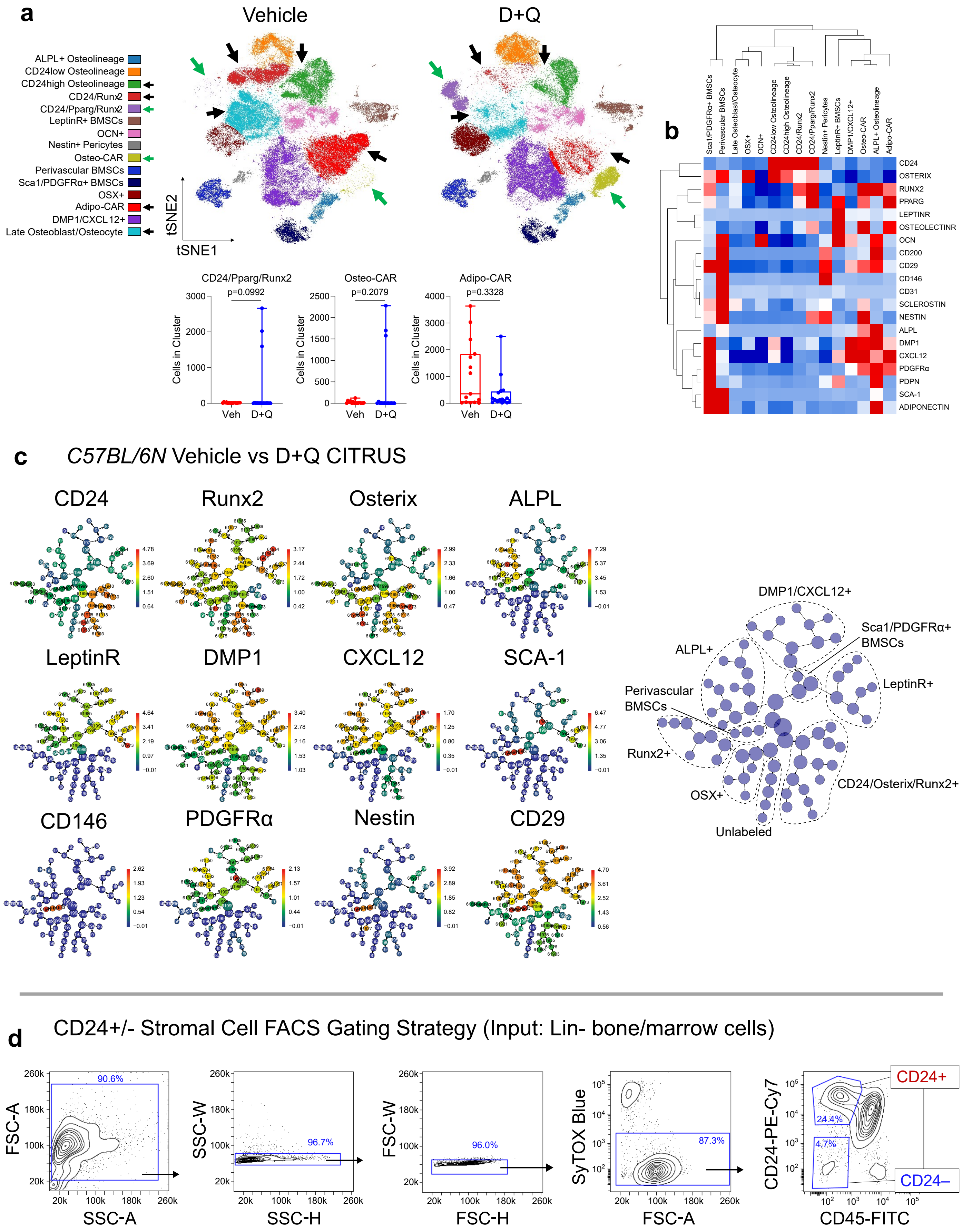
a
Vehicle
D+Q
ALPL+ Osteolineage
CD24low Osteolineage
CD24high Osteolineage
CD24/Runx2
CD24/Pparg/Runx2
LeptinR+ BMSCs
OCN+
Nestin+ Pericytes
Osteo-CAR
Perivascular BMSCs
Sca1/PDGFRα+ BMSCs
OSX+
Adipo-CAR
DMP1/CXCL12+
Late Osteoblast/Osteocyte
tSNE2
tSNE1
CD24/Pparg/Runx2
p=0.0992
Osteo-CAR
p=0.2079
Adipo-CAR
p=0.3328
Cells in Cluster
Veh
D+Q
b
CD24
OSTERIX
RUNX2
PPARG
LEPTINR
OSTEOLECTINR
OCN
CD200
CD29
CD146
CD31
SCLEROSTIN
NESTIN
ALPL
DMP1
CXCL12
PDGFRα
PDPN
SCA-1
ADIPONECTIN
c
C57BL/6N Vehicle vs D+Q CITRUS
CD24
Runx2
Osterix
ALPL
LeptinR
DMP1
CXCL12
SCA-1
CD146
PDGFRα
Nestin
CD29
DMP1/CXCL12+
Sca1/PDGFRα+ BMSCs
ALPL+
LeptinR+
Perivascular BMSCs
Runx2+
OSX+
CD24/Osterix/Runx2+
Unlabeled
d
CD24+/- Stromal Cell FACS Gating Strategy (Input: Lin- bone/marrow cells)
FSC-A
SSC-A
90.6%
SSC-W
SSC-H
96.7%
FSC-W
FSC-H
96.0%
SyTOX Blue
87.3%
CD24-PE-Cy7
CD45-FITC
CD24+
CD24–
24.4%
4.7%

Supplementary Figure 9. CyTOF analyses of aged mice undergoing D+Q Treatment. (a) t-SNE visualization and FlowSOM clustering of bone/bone marrow cells from vehicle- or D+Q-treated old mice (n=15 vehicle, n=16 D+Q; n= 5,980 cells per mouse). Clusters visually cleared are marked by black arrows, and emerging clusters are marked by green arrows. Box plots below depict clusters marked by arrows yet were not significantly cleared (Mann-Whitney test); (b) Heatmap representation of the cell clusters and protein expression of identification markers; (c) Expression plots for defining markers of CITRUS cluster families for C57BL/6N vehicle vs D+Q analyses corresponding to Figure 5d. (d) Gating strategy for isolation of live (SYTOX-) CD45-CD24+ and – cells by FACS. Box plots show median and interquartile range with error bars representing minimum and maximum values.

Supplementary Table 1. Assessment of sex as a biological variable. 2-way ANOVA results found no substantial interaction effects of sex on primary endpoints of aging and senolytic treatment.

Metric	Source of Variation	% of total variation	P value	P value summary	Significant ?
p16 median expression	Interaction	4.718	0.0846	ns	No
	Sex	0.01463	0.9209	ns	No
	**Age**	**52.86**	**<0.0001**	****	**Yes**
% p16+ cells	Interaction	5.05	0.1671	ns	No
	Sex	5.481	0.1508	ns	No
	**Age**	**31.37**	**0.0017**	**	**Yes**
% p16KB cells	Interaction	5.304	0.1346	ns	No
	Sex	8.761	0.0582	ns	No
	**Age**	**35.29**	**0.0006**	***	**Yes**
% p16KB cells	Interaction	2.326	0.4334	ns	No
	Sex	0.3027	0.7761	ns	No
	**Senolytic (AP)**	**21.14**	**0.0253**	*	**Yes**
% CD24high osteolineage cells	Interaction	1.439	0.4861	ns	No
	Sex	1.056	0.5502	ns	No
	**Senolytic (AP)**	**37.29**	**0.0016**	**	**Yes**

Supplementary Table 2. qPCR primer sequences.

Gene	Forward Primer Sequence	Reverse Primer Sequence
Actb	AATCGTGCGTGACATCAAAGAG	GCCATCTCCTGCTCGAAGTC
Gapdh	GGGAAGCCCATCACCATCTT	GCCTCACCCCATTTGATGTT
Cdkn2a (p16ink4a)	GAACTCTTTCGGTCGTACCC	AGTTCGAATCTGCACCGTAGT
Cdkn1a (p21)	GAACATCTCAGGGCCGAAAA	TGCGCTTGGAGTGATAGAAATC
Cd24a (CD24)	TGCTTCTGGCACTGCTCCTA	CGGTGCAACAGATGTTTGGTT

Supplementary Table 3. Key Resources Table

Key Resources Table		
**Reagent or resource**	**Source or reference**	**Identifiers**
**CyTOF Antibodies**		
CD45 (30-F11) - 25µg/mL	Standard BioTools	3089005B
CD146 (ME-9F1) - 50µg/mL	Standard BioTools	3141016B
LeptinR (Goat polyclonal) - 50µg/mL	R&D Systems	AF497
Nestin (Rat-401) - 25µg/mL	Biolegend	655102
Ly-6A/Sca-1 (D7) - 25µg/mL	Standard BioTools	3169015B
CD24 (M1/69) - 50µg/mL	Biolegend	101802
CD140a / PDGFRα (APA5) - 50µg/mL	BioLegend	135902
SDF-1 / CXCL12 (79018) - 50µg/mL	R&D Systems	MAB350-100
Osteolectin/Itga11 (Rabbit polyclonal) - 50µg/mL	Abcam	ab198826
CD200 (OX-90) - 50µg/mL	Biolegend	123802
CD29 (HMβ1-1) - 50µg/mL	Biolegend	102235
Runx2 (2B9) - 50µg/mL	Abcam	ab76956
SP7 (Rabbit polyclonal) - 50µg/mL	Invitrogen	PA5-40411
ALPL (Goat polyclonal) - 50µg/mL	R&D Systems	af2910
OCN (E-6) - 25µg/mL	Santa Cruz	sc-376835
E11/Podoplanin (8.1.1) - 50µg/mL	BioLegend	127401
Dmp1 (Sheep polyclonal) - 50µg/mL	ThermoFisher	PA5-47621
Sclerostin (Rabbit polyclonal) - 50µg/mL	Abcam	ab63097
PPARy (Rabbit polyclonal) - 50µg/mL	Invitrogen	PA3-821A
Adiponectin (11H4L4) - 50µg/mL	Invitrogen	PA1-84881
FLAG (L5) - 50µg/mL	Biolegend	637301
p16 (EPR20418) - 50µg/mL	Abcam	ab232402
p21 (F-5) - 50µg/mL	Santa Cruz	sc-6246
p53 (EPR20416-124) - 50µg/mL	Abcam	ab252388
CENP-B (F-4) - 50µg/mL	Santa Cruz	sc-376283
MCP-1 (2D8) - 50µg/mL	Thermo Fisher	MA5-17040
TNFα (MP6-XT22) - 50µg/mL	Standard BioTools	3162002B
PAI-1 (1D5) - 50µg/mL	Abcam	ab125687
IL-6 (MP5-20F3) - 50µg/mL	Standard BioTools	3167003B
IL-1α (ALF-161) - 50µg/mL	Biolegend	503202
IL-1β (D6D6T) - 25µg/mL	Cell Signaling	31202
CXCL1 (48415) - 25µg/mL	R&D Systems	MAB453-500
pNFkB (93H1) - 50µg/mL	Cell Signaling	3033
pATM (EPR895) - 50µg/mL	Abcam	ab217838
BCL-2 (BCL/10C4) - 50µg/mL	Biolegend	633502
yH2A-X (N1-431) - 50µg/mL	BD Biosciences	560443
Ki67 - 50µg/mL	Biolegend	350523

**Cell Sorting Reagents**		
TotalSeq™-B0212 anti-mouse CD24 Antibody - 1/400	Biolegend	101847
CD24-PE/Cyanine7 - 1/400	Biolegend	101821
CD45-FITC - 1/400	Biolegend	103107
SYTOX Blue - 1/4,000	ThermoFisher	S34857
Annexin V-Alexa 647 - 1/20	Biolegend	640912
Lineage Depletion Kit (mouse)	Miltenyi Biotec	130-090-858
CD45 microbeads (mouse)	Miltenyi Biotec	130-052-301
**Chemicals, Peptides, and Recombinant Proteins**		
AP20187	MedChemExpress	HY-13992
Dasatinib	LC Laboratories	D-3307
Quercetin	Sigma-Aldrich	Q4951
Collagenase	Sigma-Aldrich	C9891
Liberase DL	Sigma-Aldrich	5401160001
1X RBC Lysis Buffer	ThermoFisher	00-4333-57
DPBS	ThermoFisher	14190144
DMEM	ThermoFisher	11885-076
MEM α	ThermoFisher	A1049001
Opti-MEM	ThermoFisher	31985062
Fetal Bovine Serum (FBS)	Gemini Bio-Products	GEM100-106
Antibiotic-Antimycotic	ThermoFisher	15240096
Gentamicin	Sigma-Aldrich	G1397
Ascorbic Acid	Sigma-Aldrich	A8960
Beta-Glycerophosphate	Sigma-Aldrich	G9422
Bovine Serum Albumin	Sigma-Aldrich	A7906
Alizarin Red	Millipore	TMS008C
1-Step NBT/BCIP Substrate Solution (ALP)	ThermoFisher	34042
Crystal Violet	Sigma-Aldrich	C0775
DAPI ProLong Diamond Antifade Mountant	ThermoFisher	P36961
FuGENE 6 Transfection Reagent	Promega	E2691
Trypsin-EDTA (0.5%), no phenol red	Gibco	15400054
Etoposide	Sigma-Aldrich	341205
DMSO	Sigma-Aldrich	D8418
QIAzol Lysis reagent	Qiagen	79306
Annexin V Binding Buffer	Biolegend	422201
**Critical Commercial Assays**		
Maxpar X8 Antibody Labeling Kit	Standard BioTools	201149A
Cell-ID 20-Plex Pd Barcoding Kit	Standard BioTools	201060
Cell-ID Intercalator-Ir	Standard BioTools	201192B
Cell-ID Intercalator-Rh	Standard BioTools	201103A
EQ Four Element Calibration Beads	Standard BioTools	201078
Senescence β-Galactosidase Staining Kit	Cell Signaling Technology	9860

Chromium Next GEM Single Cell 3' GEM, Library & Gel Bead Kit v3.1	10X Genomics	PN-1000128
Qubit dsDNA HS and BR Assay Kits	ThermoFisher	Q32851
**Software and algorithms**		
Cytobank	Cytobank Inc.	https://premium.cytobank.org
R v4.03	R Project for Statistical Computing	https://www.r-project.org
Seurat v4.0	[120]	https://satijalab.org/seurat
Nebulosa v3.16	[122]	https://github.com/powellgenomicslab/Nebulosa
CytoTree v1.0.3	[119]	https://github.com/JhuangLab/CytoTree
CellChat v1.6.0	[121]	https://github.com/sqjin/CellChat
GraphPad Prism 8	GraphPad	http://www.graphpad.com/scientific-software/prism
ImageJ	NIH	https://imagej.nih.gov/ij
**Recombinant DNA**		
pcDNA-5/TO-p16ink4a	This paper	N/A
pCMV6-Cdkn1a	Origene	MR227529
pCMV6-Bcl-2	Origene	MR226785
pCMV6-Bglap (Ocn)	Origene	MR226351
pCMV6-Dmp1	Origene	MR221826
pCMV6-Sost	Origene	MR222588
pCMV6-Pdpn	Origene	MR201468
**Experimental Models: Organisms/Strains/Cell lines**		
INK-ATTAC	[12]	N/A
C57BL/6N	Charles River Laboratories	RRID:IMSR_CRL:027
Alb.B6.Cdkn2a-Luciferase ($p16^{Luc}$)	[38]	RRID:IMSR_NCIMR:01XBT
Runx2-Cre	[110]	
Ai9 TdTomato	Jackson Laboratory	007909
U2OS Cell Line	ATCC	ATCC (HTB-96); RRID:CVCL_0042
