# Supplementary material for: Multiparametric senescent cell phenotyping reveals targets of senolytic therapy in the aged murine skeleton
Source: Nat Commun. 2023 Jul 31;14:4587. doi: 10.1038/s41467-023-40393-9 (PMC10390564; doi:10.1038/s41467-023-40393-9)
Supplement: Supplementary file 3 — Reporting Summary [file 41467_2023_40393_MOESM3_ESM.pdf]

nature portfolio

Corresponding author(s): Sundeep Khosla

Last updated by author(s): Jul 13, 2023

Reporting Summary

Nature Portfolio wishes to improve the reproducibility of the work that we publish. This form provides structure for consistency and transparency in reporting. For further information on Nature Portfolio policies, see our Editorial Policies and the Editorial Policy Checklist.

Statistics

For all statistical analyses, confirm that the following items are present in the figure legend, table legend, main text, or Methods section.

n/a	Confirmed	
☐	☒	The exact sample size (*n*) for each experimental group/condition, given as a discrete number and unit of measurement
☐	☒	A statement on whether measurements were taken from distinct samples or whether the same sample was measured repeatedly
☐	☒	The statistical test(s) used AND whether they are one- or two-sided<br>*Only common tests should be described solely by name; describe more complex techniques in the Methods section.*
☐	☒	A description of all covariates tested
☐	☒	A description of any assumptions or corrections, such as tests of normality and adjustment for multiple comparisons
☐	☒	A full description of the statistical parameters including central tendency (e.g. means) or other basic estimates (e.g. regression coefficient) AND variation (e.g. standard deviation) or associated estimates of uncertainty (e.g. confidence intervals)
☐	☒	For null hypothesis testing, the test statistic (e.g. *F*, *t*, *r*) with confidence intervals, effect sizes, degrees of freedom and *P* value noted<br>*Give P values as exact values whenever suitable.*
☒	☐	For Bayesian analysis, information on the choice of priors and Markov chain Monte Carlo settings
☒	☐	For hierarchical and complex designs, identification of the appropriate level for tests and full reporting of outcomes
☒	☐	Estimates of effect sizes (e.g. Cohen's *d*, Pearson's *r*), indicating how they were calculated

Our web collection on statistics for biologists contains articles on many of the points above.

Software and code

Policy information about availability of computer code

Data collection	CyTOF data was collected and normalized using Cytof V6.7.1014. Flow cytometry data was collected using BD FACSDiva v9.0
Data analysis	Cytobank V9.1, Graphpad Prism 8, CellRanger v6.0, R v4.0.3 (packages: CytoTree v1.0.3, Seurat v4.0, Nebulosa v3.16, CellChat v1.6.0), ImageJ v1.54

For manuscripts utilizing custom algorithms or software that are central to the research but not yet described in published literature, software must be made available to editors and reviewers. We strongly encourage code deposition in a community repository (e.g. GitHub). See the Nature Portfolio guidelines for submitting code & software for further information.

Data

Policy information about availability of data

All manuscripts must include a data availability statement. This statement should provide the following information, where applicable:

- Accession codes, unique identifiers, or web links for publicly available datasets
- A description of any restrictions on data availability
- For clinical datasets or third party data, please ensure that the statement adheres to our policy

The CyTOF data generated in this study have been deposited in the Mendeley database under DOI: 10.17632/hg7sd7hbk5.2 [https://data.mendeley.com/datasets/hg7sd7hbk5/draft?a=3146d51a-81ab-4453-a602-79dc390f522e]. The scRNA-seq and CITE-seq data generated in this study have been deposited in the Gene Expression Omnibus (GEO) under accessions GSE237307 [https://www.ncbi.nlm.nih.gov/geo/query/acc.cgi?acc=GSE237307] and GSE237301 [https://www.ncbi.nlm.nih.gov/geo/query/acc.cgi?acc=GSE237301], respectively . Source data are provided with this paper.

Human research participants

Policy information about studies involving human research participants and Sex and Gender in Research.

Reporting on sex and gender	N/A
Population characteristics	N/A
Recruitment	N/A
Ethics oversight	N/A

Note that full information on the approval of the study protocol must also be provided in the manuscript.

Field-specific reporting

Please select the one below that is the best fit for your research. If you are not sure, read the appropriate sections before making your selection.

☒ Life sciences ☐ Behavioural & social sciences ☐ Ecological, evolutionary & environmental sciences

For a reference copy of the document with all sections, see nature.com/documents/nr-reporting-summary-flat.pdf

Life sciences study design

All studies must disclose on these points even when the disclosure is negative.

Sample size	Sample sizes were determined based on previously conducted and published experiments (PMID: 28825716, 34617510) in which statistically significant differences were observed among various senescence and skeletal parameters in response to aging or senolytic treatment.
Data exclusions	No data were excluded from analyses in this study.
Replication	For all experiments, each replicate indicates a distinct mouse sample and are described in the figure legends and methods.
Randomization	All animals were randomized by weight into treatment groups.
Blinding	All animal treatments, collections, and (where feasible) data analyses were performed in a blinded fashion.

Reporting for specific materials, systems and methods

We require information from authors about some types of materials, experimental systems and methods used in many studies. Here, indicate whether each material, system or method listed is relevant to your study. If you are not sure if a list item applies to your research, read the appropriate section before selecting a response.

March 2021

Materials & experimental systems

n/a	Involved in the study
	☒ Antibodies
	☒ Eukaryotic cell lines
☒	☐ Palaeontology and archaeology
	☒ Animals and other organisms
☒	☐ Clinical data
	☒ Dual use research of concern

Methods

n/a	Involved in the study
☒	☐ ChIP-seq
	☒ Flow cytometry
☒	☐ MRI-based neuroimaging

Antibodies

Antibodies used

CD45 (30-F11) Fluidigm 3089005B
CD146 (ME-9F1) Fluidigm 3141016B
LeptinR (Goat polyclonal) R&D Systems AF497
Nestin (Rat-401) Biolegend 655102
Ly-6A/Sca-1 (D7) Fluidigm 3169015B
CD24 (M1/69) Biolegend 101802
CD140a / PDGFRα (APA5) BioLegend 135902
SDF-1 / CXCL12 (79018) R&D Systems MAB350-100
Osteolectin/Itga11 (Rabbit polyclonal) Abcam ab198826
CD200 (OX-90) Biolegend 123802
CD29 (HMβ1-1) Biolegend 102235
Runx2 (2B9) Abcam ab76956
SP7 (Rabbit polyclonal) Invitrogen PA5-40411
ALPL (Goat polyclonal) R&D Systems af2910
OCN (E-6) Santa Cruz sc-376835
E11/Podoplanin (8.1.1) BioLegend 127401
Dmp1 (Sheep polyclonal) ThermoFisher PA5-47621
Sclerostin (Rabbit polyclonal) Abcam ab63097
PPARγ (Rabbit polyclonal) Invitrogen PA3-821A
Adiponectin (11H4L4) Invitrogen PA1-84881
FLAG (L5) Biolegend 637301
p16 (EPR20418) Abcam ab232402
p21 (F-5) Santa Cruz sc-6246
p53 (EPR20416-124) Abcam ab252388
CENP-B (F-4) Santa Cruz sc-376283
MCP-1 (2D8) Thermo Fisher MA5-17040
TNFα (MP6-XT22) Fluidigm 3162002B
PAI-1 (1D5) Abcam ab125687
IL-6 (MP5-20F3) Fluidigm 3167003B
IL-1α (ALF-161) Biolegend 503202
IL-1β (D6D6T) Cell Signaling 31202
CXCL1 (48415) R&D Systems MAB453-500
pNFkB (93H1) Cell Signaling 3033
pATM (EPR895) Abcam ab217838
BCL-2 (BCL/10C4) Biolegend 633502
γH2A-X (N1-431) BD Biosciences 560443
Ki67 (B56) Biolegend 350523
TotalSeq™-B0212 anti-mouse CD24 Antibody

Cell Sorting Reagents
CD24-PE/Cyanine7 Biolegend 101821
CD45-FITC Biolegend 103107
SYTOX Blue ThermoFisher S34857
Lineage Depletion Kit (mouse) Miltenyi Biotec 130-090-858
CD45 microbeads (mouse) Miltenyi Biotec 130-052-301
Annexin V-Alexa 647 Biolegend 640912

Validation

Refer to Table 1 for CyTOF antibody validations. In brief:
Antibodies to CD45, CD146, LeptinR, Sca-1, CXCL12, ALPL, TNFa, IL-6, and Ki67 were approved by the manufacturer for CyTOF.
Antibodies to CD24, CD140a/PDGFRa, CD200, CD29, Runx2, PDPN, Sclerostin, Pparg, Adiponectin, FLAG, MCP-1, PAI-1, IL-1B, pNFkB, p21, p16, p53, γH2A-X, and pATM were approved by the manufacturer for flow cytometry.
Antibodies to OCN, Dmp1, PDPN, Sclerostin, FLAG, p21, p16, and BCL-2 were validated for CyTOF in-house using expression constructs.
Antibodies to FLAG, MCP-1, TNFa, PAI-1, IL-6, IL-1a, IL-1B, CXCL1, pNFkB, CENP-B, p21, p16, p53, γH2A-X, pATM, and BCL-2 were

validated in-house for CyTOF using etoposide-induced senescent in vitro cultures of bone marrow stromal cells.
Antibodies to Runx2, SP7/Osterix, ALPL, and OCN were validated in-house for CyTOF using digested bone samples versus marrow-only samples.

Eukaryotic cell lines

Policy information about cell lines and Sex and Gender in Research

Cell line source(s)	U2OS (ATCC - HTB-96), Primary BMSCs (derived from digested murine bone and marrow).
Authentication	None of the cell lines used were authenticated.
Mycoplasma contamination	None of the cell lines used were tested for mycoplasma contamination.
Commonly misidentified lines (See ICLAC register)	Cell lines that were generally misidentified were not used in this study.

Animals and other research organisms

Policy information about studies involving animals; ARRIVE guidelines recommended for reporting animal research, and Sex and Gender in Research

Laboratory animals	INK-ATTAC mice (maintained on a C57BL/6N background) were collected at ages 6-month and 24-month. C57BL/6N ("Wild-Type") mice were collected at ages 6-month and 24-month. Alb.B6.Cdkn2a-Luciferase (p16Luc) mouse was collected at 6 months of age. Runx2-Cre x TdTomato mice were collected at 6-months of age. Mice were housed in ventilated cages and maintained within a pathogen-free, accredited facility under a twelve-hour light/dark cycle with constant temperature (23°C) and access to food and water ad libitum.
Wild animals	No wild animals were used.
Reporting on sex	All animal sexes are reported in the methods. Sex was considered as a biological variable (See Supplementary table 1), yet no significant interaction between sex and our primary outcomes was observed.
Field-collected samples	No field-collected samples were used in this study.
Ethics oversight	All animal studies were performed under protocols approved by the Institutional Animal Care and Use Committee (IACUC), and experiments were performed in accordance with Mayo Clinic IACUC guidelines.

Note that full information on the approval of the study protocol must also be provided in the manuscript.

Dual use research of concern

Policy information about dual use research of concern

Hazards

Could the accidental, deliberate or reckless misuse of agents or technologies generated in the work, or the application of information presented in the manuscript, pose a threat to:

No	Yes	
☒	☐	Public health
☒	☐	National security
☒	☐	Crops and/or livestock
☒	☐	Ecosystems
☒	☐	Any other significant area

Experiments of concern

Does the work involve any of these experiments of concern:

No	Yes	
☒	☐	Demonstrate how to render a vaccine ineffective
☒	☐	Confer resistance to therapeutically useful antibiotics or antiviral agents
☒	☐	Enhance the virulence of a pathogen or render a nonpathogen virulent
☒	☐	Increase transmissibility of a pathogen
☒	☐	Alter the host range of a pathogen
☒	☐	Enable evasion of diagnostic/detection modalities
☒	☐	Enable the weaponization of a biological agent or toxin
☒	☐	Any other potentially harmful combination of experiments and agents

Flow Cytometry

Plots

Confirm that:

☒ The axis labels state the marker and fluorochrome used (e.g. CD4-FITC).

☒ The axis scales are clearly visible. Include numbers along axes only for bottom left plot of group (a 'group' is an analysis of identical markers).

☒ All plots are contour plots with outliers or pseudocolor plots.

☒ A numerical value for number of cells or percentage (with statistics) is provided.

Methodology

Sample preparation	Mice were euthanized according to standardized and approved IACUC protocols. Femurs and tibiae were isolated, cleaned of soft tissue, cut at both ends, and marrow centrifuged out of the diaphyses and metaphyses into a collection tube. Marrow was resuspended in 1mg/mL Liberase DL (Sigma) diluted in FACS buffer (0.5% BSA [Sigma] in PBS) and digested at 37°C for 30 minutes to increase yield of stromal cells released from the vasculature fraction as previously described 33. Diaphyses and metaphyses cleared of bone marrow were gently crushed, rinsed in PBS, and then digested in 300 Units/mL of Collagenase IA (Sigma), diluted in MEM α (ThermoFisher), 3 times for 25 minutes each. Bone and marrow solutions were then combined and treated with RBC lysis buffer (ThermoFisher) to clear erythrocytes. The sample was then depleted of cells expressing hematopoietic lineage markers (CD5, CD45R [B220], CD11b, Gr-1 [Ly-6G/C], 7-4, and Ter-119) using Magnet Assisted Cell Sorting (MACS) and the Lineage Cell Depletion Kit (Miltenyl Biotec). Samples were incubated with anti-mouse CD45-FITC (and CD24-PECy7 for CD24- and + cell isolation) at 1:400 dilution in FACS buffer at 4°C for 20 minutes in the dark. Cells were then incubated with SYTOX blue at 1:4,000 for 5 minutes, spun down at 300xg for 5 minutes at 4°C, then resuspended in FACS buffer at 1x107 cells/mL
Instrument	FACS Aria II (BD Biosciences)
Software	BD FACSDIVA and BD Cytometer Setup and Tracking Software
Cell population abundance	CD45-CD24+: 28%. CD45-CD24-: 5%

Gating strategy	FSC-A-positive/SSC-A-positive -> SSC-W-negative -> FSC-W-negative -> SyTOX-negative -> CD45-negative/CD24-positive or -negative

☒ Tick this box to confirm that a figure exemplifying the gating strategy is provided in the Supplementary Information.
